# Clinical efficacy of endostar combined with chemotherapy in the treatment of peritoneal carcinomatosis in gastric cancer: results from a retrospective study

**DOI:** 10.18632/oncotarget.19989

**Published:** 2017-08-07

**Authors:** Jing Yao, Li Fan, Chunfen Peng, Ai Huang, Tao Liu, Zhenyu Lin, Qin Yang, Tao Zhang, Hong Ma

**Affiliations:** ^1^ Cancer Center, Union Hospital, Tongji Medical College, Huazhong University of Science and Technology, Wuhan 430022, China

**Keywords:** gastric cancer, peritoneal carcinomatosis, endostar, systemic chemotherapy

## Abstract

Peritoneal carcinomatosis (PC) resulting from metastatic dissemination of gastric cancer (GC) cells carries a dismal prognosis, and current treatments have shown little efficacy. This study aimed to evaluate the efficacy and safety of recombinant human endostatin (Endostar), a broad-spectrum anti-angiogenic peptide, in combination with chemotherapy in PC derived from GC. From January 2014 to December 2016, 33 patients with advanced stage GC associated with PC were enrolled. Pathological, imaging, and treatment data were retrospectively analyzed. Twenty-one patients received systemic chemotherapy (control group), while 12 patients were administered Endostar and chemotherapy. Combined treatment with Endostar/chemotherapy showed the tendency to increase objective response rate (41.7% vs. 23.8%) and disease control rate (83.3% vs. 61.9%) compared with the control group, although the differences were not statistically significant. Endostar plus chemotherapy effectively extended time to progression (4.6 ± 0.3 months vs. 3.5 ± 0.3 months, P = 0.03) and median overall survival (15.8 ± 1.7 months vs. 9.8 ± 0.9 months, P = 0.01) compared with chemotherapy alone. The combination therapy did not cause more adverse reactions than chemotherapy alone. Thus, the addition of Endostar to conventional chemotherapy treatment effectively attenuated the development of PC and extended survival, with high safety and tolerance.

## INTRODUCTION

Once peritoneal carcinomatosis (PC) occurs in gastric cancer (GC) patients, prognosis becomes extremely poor [1]. Although many chemotherapeutics, comprising currently four categories that include six drugs, have been developed for GC, the therapeutic outcome is still not ideal. The 5-year survival rate in advanced GC is only 20%, whereas the 2-year survival rate of GC patients with combined PC is less than 5%. Furthermore, median survival hardly exceeds one year [2]. Targeted drugs provide new hope for the treatment of various malignant tumors. Current drugs for GC include products affecting proliferation-related signaling pathways and the tumor microenvironment. The former group includes trastuzumab, an antibody that binds epidermal growth factor receptor (EGFR) and blocks its downstream signaling pathway. The Trastuzumab for Gastric Cancer (ToGA) study indicated that trastuzumab prolonged survival time in HER-2 positive patients. However, the HER-2 amplification rate in GC is only ∼15%, and thus most patients would not benefit from the antibody. Moreover, the ToGA study did not assess therapeutic efficacy for PC, hence the benefit of anti-HER-2 therapy in GC patients with PC remains unclear [3]. Another antibody, bevacizumab, blocks tumor angiogenesis by binding to vascular endothelial growth factor (VEGF). Although therapy with bevacizumab showed no efficacy in GC patients, both preclinical and clinical studies indicated that anti-VEGF therapy is effective in reducing ascitic cancerous exudates in GC and other tumors [4, 5]. Therefore, anti-angiogenesis remains promising in the treatment of advanced GC, especially in cases with PC.

Endostatin is an endogenous angiogenesis inhibitor, widely applied in clinic, that inhibits tumor growth by various mechanisms. Folkman summarized about 160 reports assessing endostatin in decades ago (1997-2006), and found that it affects the expression of 12% of the genes in endothelial cells. Upregulated genes included those encoding angiogenic inhibitors, while downregulated genes comprised those associated with pro-angiogenic factors, indicating that endostatin has strong anti-angiogenic actions [6]. Endostar is a recombinant endostatin with nine additional amino acids, which increases the stability and solubility of the molecule and enhances its anti-angiogenic effects [7]. *In vivo* studies found that Endostar inhibited tumor growth in nude mouse models of GC, reduced the expression of pro-angiogenic factors such as VEGF and basic fibroblast growth factor (bFGF), and induced tumor cell apoptosis [8-10]. Endostar in combination with chemotherapy was also reported to effectively inhibit the formation of malignant serous effusion by transplantation tumors in nude mice, effectively decreasing the number of peritoneal tumor nodules in a model of PC from GC [11, 12]. A clinical exploratory research with a small sample size indicated the effectiveness and feasibility of Endostar in combination with chemotherapy in the treatment of advanced GC [13]. However, whether GC patients with PC may benefit from the above combinatory therapy remains unknown. Therefore, the present study assessed the effectiveness and safety of Endostar combined with conventional chemotherapy in patients with advanced GC and PC.

## RESULTS

### Patient baseline characteristics

From January 2014 to December 2016, a total of 33 patients with advanced GC combined with PC were enrolled, and their pathological, imaging, and treatment data were retrospectively analyzed. Twenty-one patients received systemic chemotherapy, while 12 received Endostar in combination with chemotherapy. All 33 patients were evaluated for phase-efficacy and followed-up for survival time assessment. The median age of all patients was 54 years old, and no significant differences in patient performance status score, tumor differentiation, and tumor staging (such as T and N stage) were noted between the two groups. Given that PC associated with metastases to other organs might affect prognosis, the metastatic sites were classified as ‘single PC’ or ‘organ-associated PC’. Metastatic sites were uniformly distributed, and no intergroup differences were noted. Analysis of previous therapeutic factors (e.g. surgery and adjuvant chemotherapy) indicated that 3 cases in the combination therapy group had received radical surgery and 2 cases received adjuvant chemotherapy, and then developed PC. In the chemotherapy group, 4 patients underwent radical surgery and received adjuvant chemotherapy, then presented with PC. No significant differences in these parameters were observed for the two groups. The patients’ clinical characteristics are summarized in Table 1.

**Table 1:** Patients’ clinic pathologic characteristics

Patient characteristics	Combined group N (%)	Control group N (%)	P value
Median years (range)	54 (34-65)	54 (27-69)	
Gender			0.74
Male	7 (58.3)	11 (52.4)	
Female	5 (41.7)	10 (47.6)	
Performance status			0.86
0-1	10 (83.3)	17 (80.95)	
2	2 (16.7)	4 (19.1)	
Histology			0.44
Well/moderately differentiated	3 (25.0)	3 (14.3)	
Poorly differentiated	9 (75.0)	18 (85.7)	
T stage			0.63
T1-2	2 (16.7)	5 (23.8)	
T3-4	10 (83.3)	16 (76.2)	
N stage			0.90
N0	1 (8.3)	2 (9.5)	
N+	11 (91.7)	19 (90.5)	
Metastasis sites status			0.55
Peritoneal carcinomatosis (PC)	7 (58.3)	10 (47.6)	
PC + other metastasis	5 (41.7)	11 (52.4)	
Previous treatment			
Surgery	3 (25.0)	4 (19.1)	0.68
Adjuvant chemotherapy	2 (16.7)	4 (19.1)	0.86

### Treatment efficacy and survival analysis

All 33 patients received at least 2 cycles (average = 5 cycles) of 21 days of therapy, and qualified for efficacy evaluation. In the combination therapy group, 9 cases (75%) mainly used oxaliplatin as chemotherapeutic, while 3 cases (25%) mainly employed irinotecan or docetaxel. In the control group these rates were 76.2% and 23.8%, respectively, with no significant differences registered between the two groups. Efficacy evaluation was conducted for each patient every 2 cycles. In the combination therapy group, 1 patient achieved complete response (CR), 4 achieved partial response (PR), 5 had stable disease (SD), and 2 showed progressive disease (PD) after 2 treatment cycles. Objective response rate (ORR) and disease control rate (DCR) were 41.7% and 83.3%, respectively. In the control group, no patient achieved CR, 5 achieved PR, 8 had SD, and 8 manifested PD after 2 cycles. ORR and DCR were 23.8% and 61.9%, respectively. Although no statistically significant differences between the two groups were obtained, these findings indicated that treatment with Endostar combined with chemotherapy still exhibited the tendency to increase ORR and DCR compared with the control group (Table 2).

**Table 2:** Summary of treatment administration and treatment response

Treatment administration	Combined group (N=12)	Control group (N=21)	P value
Chemotherapy regimens			0.93
OXA	9 (75.0%)	16 (76.2%)	
CPT-11 / Taxol	3 (25.0%)	5 (23.8%)	
Chemotherapy cycle			
< 4cycles	5 (41.6%)	15 (65.3%)	0.38
> 4cycles	7 (58.4%)	8 (34.7%)	
Treatment response			
Complete response, CR	1	0	
Partial response, PR	4	5	
Stable disease, SD	5	8	
Progression disease, PD	2	8	
ORR	41.7%	23.8%	0.28
DCR	83.3%	61.9%	0.19
Median TTP(m)	4.6 ± 0.3	3.5 ± 0.3	0.03
Median OS (m)	15.8 ± 1.7	9.8 ± 0.9	0.01

ORR, objective response rate; DCR, disease control rate; TTP, Time to tumor progression; OS, overall survival; m, month; N, number of samples in each group.

**Figure 1:**
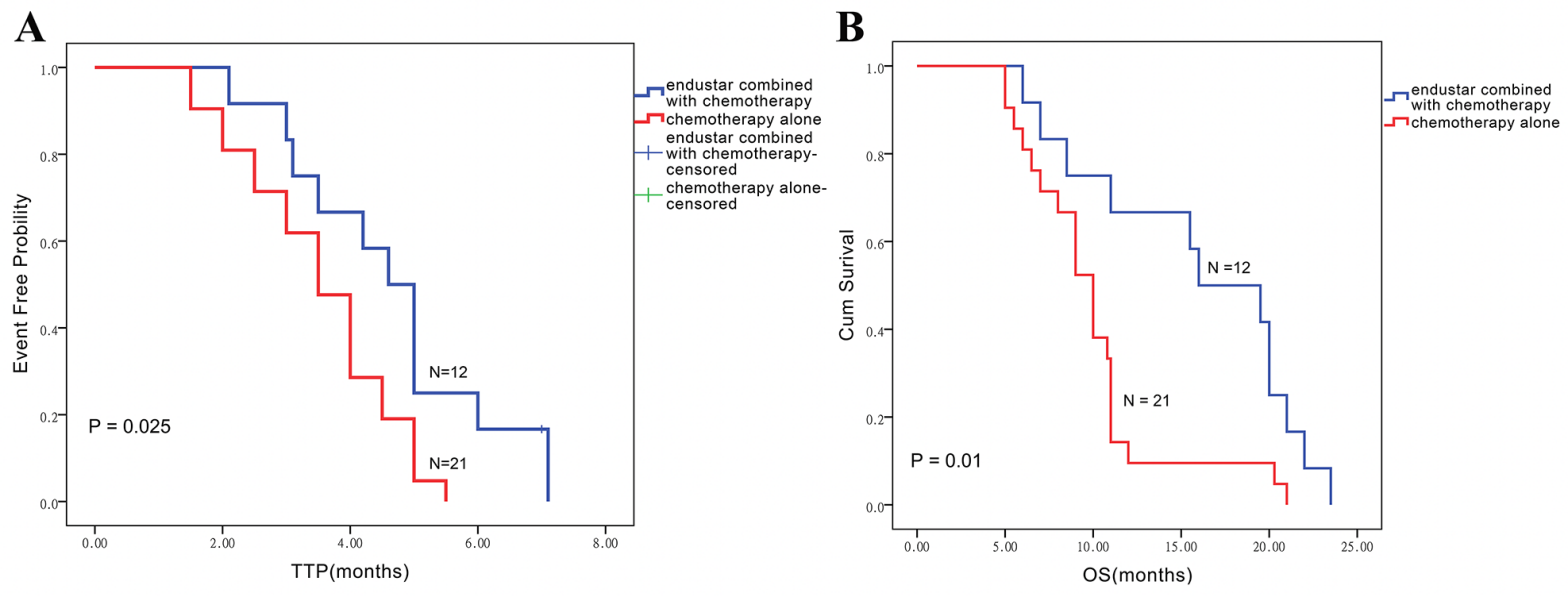
The Kaplan-Meier method was applied to assess TTP **(A)** and overall survival **(B)** in the endostar/chemotherapy combination and chemotherapy alone groups.

In this study, follow-up was performed every 3 months until disease progression or death to get the survival data as time to tumor progression (TTP) and overall survival (OS), only one case in combination group was consider as censored data for the analysis of TTP because this patient refused the subsequent treatment of Endostar after completing 6 cycles treatment and got PR in tumor response evaluation, however, this patient still was followed up to get the final data of OS. Therefore, nearly all patients have been followed-up effectively to get the survival data. The median follow-up duration was 12 months (range 5-23.5 months) for all the patients, and survival analysis indicated that Endostar combined with chemotherapy effectively extended median time of TTP, which was 4.6 ± 0.3 months (95% CI 3.9-5.3 months) in the combination group, versus 3.5 ± 0.3 months (95% CI 2.8-4.1 months) in the control group (P = 0.03). Also, Endostar combined with chemotherapy conferred an overall survival (OS) advantage, with a significantly longer median OS (15.8 ± 1.7 months, 95% CI 9.2-22.8 months) than that of the control group (9.8 ± 0.9 months, 95% CI 8.5-11.5 months) (P = 0.01) (Figure 1A-1B).

**Figure 2:**
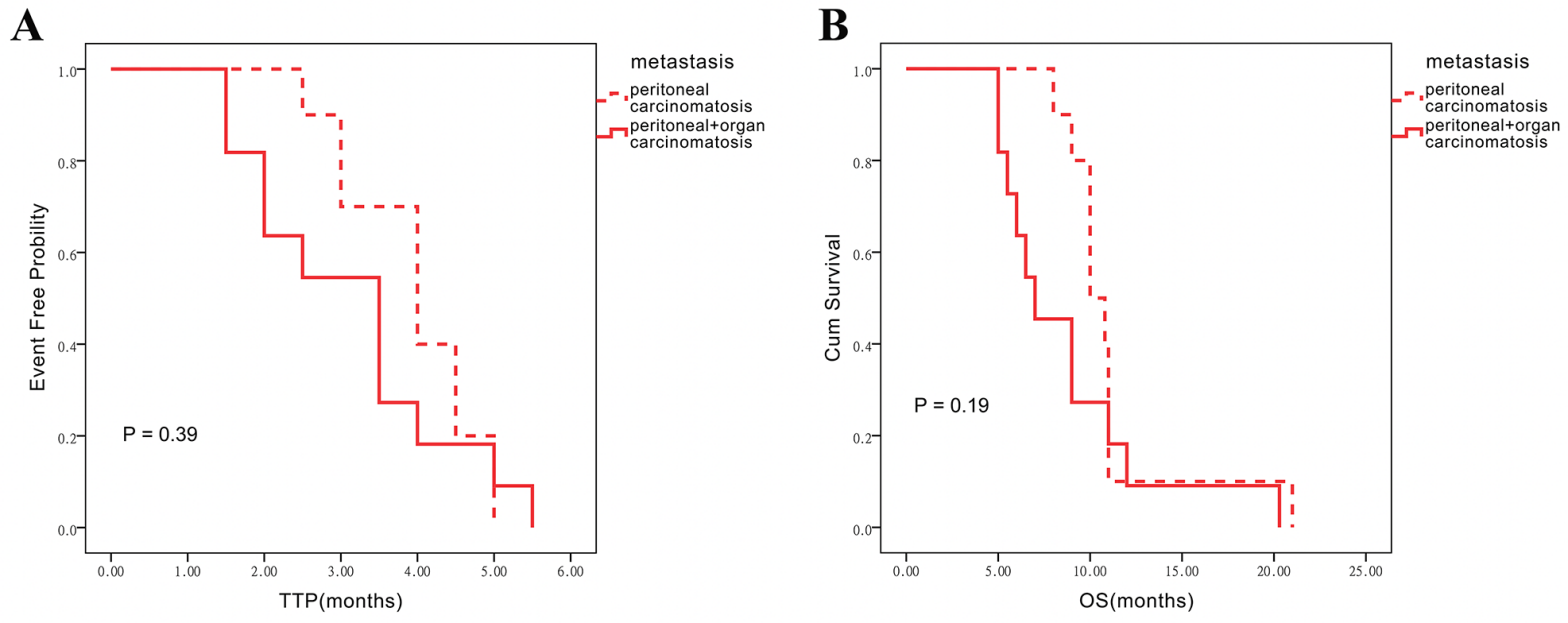
Influence of different metastatic statuses on TTP and OS assessed in the chemotherapy alone group **(A)** Influence on TTP in the chemotherapy alone group. **(B)** Influence on OS in chemotherapy alone group.

**Figure 3:**
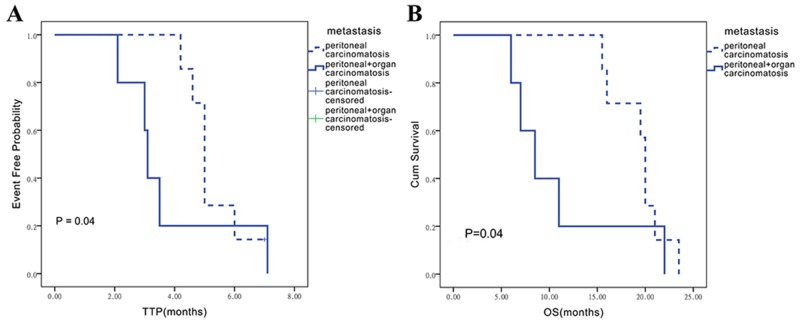
Influence of different metastatic statuses on TTP and OS assessed in the endostar/chemotherapy combination group **(A)** Influence on TTP in the endostar/chemotherapy combination group. **(B)** Influence on OS in the endostar/chemotherapy group.

The effects of metastatic statuses on TTP and OS were assessed in the control and combination group. In control group, ‘organ-associated PC’ group revealed a little shorter median TTP and OS compared with ‘single PC’ group, however no difference has been observed between two metastatic statuses (Figure 2A-2B). Interestingly, the ‘single PC’ group (n = 7) showed longer median TTP compared with the ‘organ-associated PC’ group (n = 5) (5.2 ± 0.3 vs. 3.1 ± 0.1; P = 0.04). Similarly, OS in patients with single PC administered Endostar was longer than that obtained for individuals with organ metastasis (19.3 ± 1.1 vs. 8.5 ± 1.6; P = 0.04) (Figure 3A-3B). The results seemed implied Endostar maybe have more potential in control single peritoneal carcinomatosis than complicated organ metastasis.

In addition, univariate and multivariate Cox regression analysis were performed to analysis the association of some clinical factors and OS in gastric cancer with peritoneal carcinomatosis (Table 3), it was found tumor grading, tumor metastatic status as single PC or combined with organ metastasis, number of chemotherapy cycles (< 4 cycles versus > 4 cycles) and Endostar therapy were factors influencing prognosis. In further, the metastatic status, number of chemotherapy cycles and Endostar therapy revealed the value of independent factors influencing prognosis through multivariable analysis.

**Table 3:** Univariate and multivariate Cox regression analysis of clinical factors for OS in gastric cancer with peritoneal carcinomatosis

	Univariate analysis			Multivariate analysis		
	HR	95%CI	P value	HR	95%CI	P value
Age	0.98	0.93-1.02	0.21			
Gender	0.74	0.36-1.49	0.13			
Tumor grade	0.35	0.12-1.02	0.05*	0.48	0.15-1.48	0.20
T stage	1.46	0.59-3.59	0.41			
N stage	1.11	0.34-3.68	0.86			
Status of metastatic site	2.32	1.12-4.81	0.02*	2.32	1.11-4.86	0.02*
Previous Surgery	0.93	0.39-2.16	0.87			
Adjuvant chemotherapy	1.07	0.43-2.61	0.89			
Chemotherapy regimens	0.90	0.36-2.23	0.82			
Chemotherapy cycle number ( < 4cycles VS > 4 cycles)	0.30	0.13-0.68	0.004*	0.24	0.09-0.60	0.002*
Endostar	2.63	1.16-5.97	0.02*	2.42	1.04-5.63	0.04*

HR, hazard ratio; CI, confidence interval; VS, versus, *p<0.05 statistically significant.

### Adverse events

All 33 patients completed safety evaluation (Table 4). None stopped therapy because of severe drug toxicity. Four patients showed Level 3 or Level 4 hematotoxicity manifested as neutropenia, including 1 (8.3%) in the combination therapy and 3 (14.3%) receiving chemotherapy alone; however, no patient showed febrile neutropenia. One patient (4.8%) in the chemotherapy group showed Level 4 thrombocytopenia; although the patient recovered after therapy, the following treatment cycle was delayed. Level 3 or 4 non-hematotoxicity mainly manifested as nausea, vomiting, and liver function impairment, all of which were reversed after therapy. Other toxicities, such as diarrhea and peripheral neuritis, were mild and tolerated. Four cases administered oxaliplatin therapy in both groups showed temporary allergic reactions and recovered after treatment. Three cases showed adverse reactions related to Endostar (e.g. hypertension and bleeding), which were Level 1-2 toxicities. Therefore, among all adverse events, only neutropenia and thrombocytopenia were the main reasons for delayed therapy and dosage decrease. Only 4 of the 33 patients had oxaliplatin dosage decreased by 10% due to Level 4 neutropenia and thrombocytopenia, whereas no dosage decrease was required for other chemotherapeutics, including Endostar.

**Table 4:** Treatment-related toxicities

Toxicities	Combined group		Control group		P value	
	All grades N (%)	Grade 3/4 N (%)	All grades N (%)	Grade 3/4 N (%)	All grades	Grade3/4
Hematologic						
Leukopenia	7 (58.3)	1 (8.3)	18 (85.7)	3 (14.3)	0.07	0.61
Neutropenia	7 (58.3)	1 (8.3)	18 (85.7)	3 (14.3)	0.07	0.61
Anemia	10 (83.3)	0	17 (80.9)	1 (4.8)	0.67	0.44
Thrombocytopenia	4 (33.3)	0	4 (19.1)	1 (4.8)	0.35	0.44
Non-hematologic						
Asthenia	7 (58.3)	0	16 (76.2)	1 (4.8)	0.28	0.44
Anorexia	7 (58.3)	0	16 (76.2)	1 (4.8)	0.28	0.44
Allergy	1 (8.3)	0	3 (14.3)	0	0.61	
Nausea	8 (66.7)	1 (8.3)	12 (57.1)	2 (9.5)	0.59	0.91
Vomiting	8 (66.7)	1 (8.3)	12 (57.1)	2 (9.5)	0.59	0.91
Diarrhea	2 (16.7)	0	3 (14.3)	0	0.85	
Constipation	7 (58.3)	1 (8.3)	15 (71.4)	1 (4.8)	0.44	0.67
Stomatitis	2 (16.7)	0	2 (9.5)	0	0.54	
Hyperbilirubinemia	2 (16.7)	0	7 (33.3)	1 (4.8)	0.30	0.44
Elevated AST/ALT	8 (66.7)	0	7 (33.3)	1 (4.8)	0.06	0.44
Peripheral neuropathy	7 (58.3)	0	10 (47.6)	0	0.55	
Hand-foot syndrome	5 (41.7)	0	6 (28.6)	1 (4.8)	0.44	0.44
Hypertension	3 (25.0)	0	1 (4.8)	0	0.09	
Bleeding	1 (8.3)	0	0	0	0.18	

AST, aspartate aminotransferase; ALT, alanine aminotransferase.

## DISCUSSION

GC with PC is considered an extremely serious condition with very poor prognosis. Although advances in tumor reductive surgery techniques have increased the survival of patients with PC, not all patients can receive cytoreductive surgery, and 10-40% of those who do show relapse [2]. Thus, chemotherapy remains the main treatment option for advanced GC associated with PC, even though the effectiveness rates of chemotherapeutic drugs used as monotherapy are only 14-25%, with a median survival time of 7-10 months [14]. Recently, new targeted drugs for GC have emerged, including monoclonal antibodies against VEGF, the VEGF receptor (VEGFR), and HER-2, and mammalian target of rapamycin (mTOR) inhibitors; however, no breakthrough has been achieved in treating advanced GC, associated or not with PC [15].

In 1971, Folkman found that various growth factors such as VEGF and bFGF were continuously secreted by cancer cells to promote neovascularization and induce tumor proliferation, infiltration and metastasis, providing a theoretical basis for anti-angiogenesis in tumor therapy [16]. In 1983, Senger found that VEGF and other growth factors were highly expressed in ascites and peritoneal tissue of nude mice bearing tumors [17]. These data, later supported by clinical findings, suggested that anti-tumor angiogenesis therapy could be effective for treating malignant ascites [5]. Studies showed, for instance, that anti-VEGF therapy combined with chemotherapy improved survival in patients with colorectal cancer associated with PC, compared with chemotherapy alone [18]. Similarly, better disease control was achieved with anti-VEGF therapy in patients with ovarian cancer associated with carcinomatosis [19, 20]. Meanwhile, although anti-VEGF antibody therapy failed to show effectiveness in advanced GC, a survival benefit was still obtained in second-line and third-line therapies using anti-VEGFR-2 monoclonal antibodies and small-molecule inhibitors, suggesting that some anti-angiogenic agents inducing VEGF/VEGFR signaling blockade may be effective [21, 22]. Considering also that heterogeneity within GC could result in tolerance to mono-anti-angiogenic factors, most evidence suggests that broad-spectrum anti-angiogenesis drugs may be more effective in GC treatment, especially in cases with PC.

Endostatin, the 184-amino-acid long, 20 kD C-terminal fragment of collagen XVII, was first purified from murine hemangioendothelioma strain culture medium [23]. Both *in vitro* and *in vivo* studies showed that endostatin effectively inhibits tumor proliferation by directly blocking the VEGF/VEGFR2 pathway and inhibiting VEGFR tyrosine phosphorylation. Moreover, endostatin’s broad-spectrum anti-angiogenic effects include attenuation of lymphangiogenesis by inhibition of VEGFR3 signaling [24]. Endostar is a recombinant human endostatin structurally modified to increase stability, solubility, and activity [7]. Based on clinical data showing that Endostar in combination with chemotherapy is beneficial for patients with advanced lung cancer, the State Food and Drug Administration (SFDA) of China approved its use for non-small cell lung cancer therapy in 2005, while potential applications in other tumors are under investigation [25]. GC is highly vascularized, so its occurrence and development are closely related to tumor angiogenesis. Accordingly, microvessel density as well as VEGF and endostatin expression in GC tissues are relevant to prognosis [26, 27]. Preclinical studies demonstrated that recombinant endostatin can inhibit the proliferation of GC cells, and effectively decrease malignant serosal cavity effusion as well as generation of PC nodes. Also, a preliminary clinical exploration found that combination chemotherapy with Endostar was superior than chemotherapy alone in the developmental phase of GC [28]. However, clinical data of Endostar’s effectiveness in the treatment of GC associated with PC remain limited.

To address this evidence gap, this retrospective study aimed to assess the effectiveness, safety, and survival outcomes of Endostar in combination with chemotherapy in advanced GC associated with PC. Interestingly, Endostar has been shown to effectively induce vascular normalization 5-7 days after therapy [29, 30], and to increase drug efficacy. Thus, when screening cases administered the combination therapy, Endostar administration should exceed 5 days per cycle to be effective, and continuous therapy of 2 cycles is necessary to evaluate efficacy. To properly assess efficacy, we analyzed CT/MRI imaging data to screen evaluable lesions. Based on the above requirements, 33 patients were enrolled, of which 12 were allocated to the Endostar plus chemotherapy group and 21 received chemotherapy alone. All patients received chemotherapy for at least 2 cycles. Although patients administered combination therapy evidenced increased ORR and DCR, no statistically significant differences were noted with the group of patients treated with chemotherapy alone. On the other hand, the combined treatment effectively provided a survival benefit by extending the patients’ median TTP and OS. Regression analyses indicated that tumor stage, tumor metastasis status, and Endostar therapy were factors predicting prognosis. However, only metastatic status and Endostar therapy were independent prognostic factors. Importantly, compared with chemotherapy alone, the combination therapy did not increase adverse reactions. Furthermore, anti-angiogenesis-related side effects such as hypertension and bleeding tendency were controllable. In addition, no thrombotic events or proteinuria were observed, indicating good tolerability.

PC from GC is difficult to treat, and no effective therapeutic drugs or treatment strategies are available. Our study revealed that Endostar addition greatly improves the efficacy of conventional chemotherapy in the treatment of malignant ascites, restraining the development of PC and extending survival with high safety and good tolerance. Since this was a retrospective study with a small sample size, further prospective studies are warranted to confirm Endostar’s clinical value in advanced GC with PC.

## MATERIALS AND METHODS

### Research design and patient enrollment

This retrospective observational study analyzed pathological, imaging, and treatment data from 33 patients with advanced stage GC associated with PC enrolled in Wuhan Union Hospital, Huazhong University of Science and Technology, from January 2014 to December 2016. Inclusion criteria were: age ranging from 18 to 75 years; GC diagnosed by pathology; PC confirmed by CT or MRI imaging, with at least one evaluable lesion according to the Response Evaluation Criteria in Solid Tumor (RECIST) standard [31]; up to two cycles (21 days/cycle) of first-line chemotherapy for metastatic GC received after diagnosis (with or without Endostar); efficacy evaluation completion, and traceable follow-up. Clinical characteristics, such as age, gender, physical condition, tumor differentiation, staging, metastatic sites, and treatment method (radical surgery or adjuvant chemotherapy) were recorded. All patients effectively receiving treatment were followed-up until collection of survival data was complete. This study was approved by the Ethics Committee of Huazhong University of Science and Technology.

### Treatment assignment

All enrolled patients received standard fist-line chemotherapy with or without endostar based on economic condition and inclination, were classified as combination therapy group and control group, respectively. In consideration that some previous studies have showed endostar can effectively induce vascular normalization and increase subsequent treatment efficacy administered 5∼7 days before routine therapy [29, 30], Thus, our study plan was designed to enroll the patients treated with Endostar before chemotherapy. Therapy regimen was as following: patients were administered Endostar 30 mg/d for 5 days (d1-d5) before standard first line chemotherapy; First line therapy included oxaliplatin, irinotecan, or docetaxel, repeated within 2 or 3 weeks. Patients were evaluated for treatment effectiveness according to the RECIST standard every 2 months. Efficacy evaluation included physical examination, blood count, serum biochemistry, chest imaging, and abdominal pelvic examination. Safety was evaluated at baseline, before each treatment cycle, and 4-6 weeks after the final therapy. All the patients were followed-up regularly, and efficacy was evaluated every 3 months until disease progression or death, according to the RECIST standard.

### Treatment efficacy evaluation

Tumor response to treatment was evaluated and defined as CR, PR, SD, and PD according to the RECIST guidelines. ORR was reflected by the proportion of patients with CR or PR; DCR denoted the proportion of patients with positive response of CR, PR, or SD. Meanwhile, the patients were followed-up regularly until disease progression or death, to obtain survival data as TTP (time from treatment initiation to disease progression) and OS (time from treatment initiation to death), and censored data was recorded as the day lost to follow up. The safety index was assessed based on patients who have received at least one treatment; adverse events were evaluated according to The National Cancer Institute Common Toxicity Criteria for Adverse Events (NCI-CTC AE, version 3.0) [32].

### Statistical analysis

SPSS16.0 software was used for data analysis. Chi-square test was used to assess differences in clinical pathological variables, efficacy, and toxicity between the combination therapy and chemotherapy alone groups. Data was expressed as mean ± SD. The Kaplan-Meier method was used to assess TTP and OS; COX regression was used to detect factors influencing survival through comparison between the two groups by log-rank test. P < 0.05 was considered statistically significant.

## Abbreviations

TTP: time to progression
OS: overall survival
ORR: objective response rate
DCR: disease control rate
EGFR: epidermal growth factor receptor
VEGF: vascular endothelial growth factor
bFGF: basic fibroblast growth factor
mTOR: mammalian target of rapamycin
VEGFR: VEGF receptor
SFDA: State Food and Drug Administration
RECIST: Response Evaluation Criteria in Solid Tumor
CR: complete response
PR: partial response
SD: stable disease
PD: progressive disease

## References

[1] Yonemura Y, Bandou E, Kawamura T, Endou Y, Sasaki T (2006). Quantitative prognostic indicators of peritoneal dissemination of gastric cancer. Eur J Surg Oncol.

[2] Beeharry MK, Liu WT, Yao XX, Yan M, Zhu ZG (2016). A critical analysis of the cytoreductive surgery with hyperthermic intraperitoneal chemotherapy combo in the clinical management of advanced gastric cancer: an effective multimodality approach with scope for improvement. Transl Gastroenterol Hepatol.

[3] Bang YJ, Van Cutsem E, Feyereislova A, Chung HC, Shen L, Sawaki A, Lordick F, Ohtsu A, Omuro Y, Satoh T, Aprile G, Kulikov E, Hill J (2010). Trastuzumab in combination with chemotherapy versus chemotherapy alone for treatment of HER2-positive advanced gastric or gastro-oesophageal junction cancer (ToGA): a phase 3, open-label, randomised controlled trial. Lancet.

[4] Ohtsu A, Shah MA, Van Cutsem E, Rha SY, Sawaki A, Park SR, Lim HY, Yamada Y, Wu J, Langer B, Starnawski M, Kang YK (2011). Bevacizumab in combination with chemotherapy as first-line therapy in advanced gastric cancer: a randomized, double-blind, placebo-controlled phase III study. J Clin Oncol.

[5] Kobold S, Hegewisch-Becker S, Oechsle K, Rha SY, Sawaki A, Park SR, Lim HY, Yamada Y, Wu J, Langer B, Starnawski M, Kang YK (2009). Intraperitoneal VEGF inhibition using bevacizumab: a potential approach for the symptomatic treatment of malignant ascites?. Oncologist.

[6] Folkman J (2006). Antiangiogenesis in cancer therapy--endostatin and its mechanisms of action. Exp Cell Res.

[7] Fu Y, Tang H, Huang Y, Song N, Luo Y (2009). Unraveling the mysteries of endostatin. IUBMB Life.

[8] Wang YB, Liu JH, Song ZM (2013). Effects of recombinant human endostatin on the expression of vascular endothelial growth factor in human gastric cancer cell line MGC-803. Biomed Rep.

[9] Li DN, Wang L, Wang L, Li S, Wang YB (2016). Expression of inhibitor of differentiation-1 and its effects on angiogenesis in gastric cancer. Cancer Biother Radiopharm.

[10] Guo ZY, Yao GD, Fu LP, Fu ZG, Hou B (2015). Effect of recombinant human endostatin on the expression of c-Myc and bFGF in mouse gastric cancer cells. Genet Mol Res.

[11] Jia L, Ren S, Li T, Wu J, Zhou X, Zhang Y, Wu J, Liu W (2017). Effects of combined simultaneous and sequential endostar and cisplatin treatment in a mice model of gastric cancer peritoneal metastases. Gastroenterol Res Pract.

[12] Ma X, Yao Y, Yuan D, Liu H, Wang S, Zhou C, Song Y (2012). Recombinant human endostatin endostar suppresses angiogenesis and lymphangiogenesis of malignant pleural effusion in mice. PLoS One.

[13] Gao SR, Li LM, Xia HP, Wang GM, Xu HY, Wang AR (2015). Clinical observation on recombinant human endostatin combined with chemotherapy for advanced gastrointestinal cancer. Asian Pac J Cancer Prev.

[14] Montori G, Coccolini F (2014). The treatment of peritoneal carcinomatosis in advanced gastric cancer: state of the art. Int J Surg Oncol.

[15] de Mestier L, Lardiere-Deguelte S, Volet J, Kianmanesh R, Bouché O (2016). Recent insights in the therapeutic management of patients with gastric cancer. Dig Liver Dis.

[16] Folkman J (1971). Tumor angiogenesis: therapeutic implications. N Engl J Med.

[17] Senger DR, Galli SJ, Dvorak AM, Perruzzi CA, Harvey VS, Dvorak HF (1983). Tumor cells secrete a vascular permeability factor that promotes accumulation of ascites fluid. Science.

[18] Adachi T, Hinoi T, Egi H, Shimomura M, Ohdan H (2015). Oxaliplatin and molecular-targeted drug therapies improved the overall survival in colorectal cancer patients with synchronous peritoneal carcinomatosis undergoing incomplete cytoreductive surgery. Surg Today.

[19] Kesterson JP, Mhawech-Fauceglia P, Lele S (2008). The use of bevacizumab in refractory ovarian granulosa-cell carcinoma with symptomatic relief of ascites: a case report. Gynecol Oncol.

[20] Hamilton CA, Maxwell GL, Chernofsky MR, Bernstein SA, Farley JH, Rose GS (2008). Intraperitoneal bevacizumab for the palliation of malignant ascites in refractory ovarian cancer. Gynecol Oncol.

[21] Wilke H, Muro K, Van Cutsem E, Oh SC, Bodoky G, Shimada Y, Hironaka S, Sugimoto N, Lipatov O, Kim TY, Cunningham D, Rougier P, Komatsu Y (2014). Ramucirumab plus paclitaxel versus placebo plus paclitaxel in patients with previously treated advanced gastric or gastro-oesophageal junction adenocarcinoma (RAINBOW): a double-blind, randomised phase 3 trial. Lancet Oncol.

[22] Li J, Qin S, Xu J, Guo W, Xiong J, Bai Y, Sun G, Yang Y, Wang L, Xu N, Cheng Y, Wang Z, Zheng L (2013). Apatinib for chemotherapy-refractory advanced metastatic gastric cancer: results from a randomized, placebo-controlled, parallel-arm, phase II trial. J Clin Oncol.

[23] O’Reilly MS, Boehm T, Shing Y, Fukai N, Vasios G, Lane WS, Flynn E, Birkhead JR, Olsen BR, Folkman J (1997). Endostatin: an endogenous inhibitor of angiogenesis and tumor growth. Cell.

[24] Ling Y, Yang Y, Lu N, You QD, Wang S, Gao Y, Chen Y, Guo QL (2007). Endostar, a novel recombinant human endostatin, exerts antiangiogenic effect via blocking VEGF-induced tyrosine phosphorylation of KDR/Flk-1 of endothelial cells. Biochem Biophys Res Commun.

[25] Zhao X, Su Y, You J, Gong L, Zhang Z, Wang M, Zhao Z, Zhang Z, Li X, Wang C (2016). Combining antiangiogenic therapy with neoadjuvant chemotherapy increases treatment efficacy in stage IIIA (N2) non-small cell lung cancer without increasing adverse effects. Oncotarget.

[26] Kolev Y, Uetake H, Iida S, Ishikawa T, Kawano T, Sugihara K (2007). Prognostic significance of VEGF expression in correlation with COX-2, microvessel density, and clinicopathological characteristics in human gastric carcinoma. Ann Surg Oncol.

[27] Woo IS, Kim KA, Jeon HM, Hong SH, Rho SY, Koh SJ, Lee MA, Byun JH, Kang JH, Hong YS, Lee KS, Cho CS, Choi MG, Chung IS (2006). Pretreatment serum endostatin as a prognostic indicator in metastatic gastric carcinoma. Int J Cancer.

[28] Xu R, Ma N, Wang F, Ma L, Chen R, Chen R, Kebinu M, Ma L, Han Z, Ayixiamu Mayier M, Su P, Naman Y (2013). Results of a randomized and controlled clinical trial evaluating the efficacy and safety of combination therapy with Endostar and S-1 combined with oxaliplatin in advanced gastric cancer. Onco Targets Ther.

[29] Jiang XD, Dai P, Wu J, Song DA, Yu JM (2012). Effect of recombinant human endostatin on radiosensitivity in patients with non-small-cell lung cancer. Int J Radiat Oncol Biol Phys.

[30] Jiang XD, Dai P, Qiao Y, Wu J, Song DA, Li SQ (2012). Clinical study on the recombinant human endostatin regarding improving the blood perfusion and hypoxia of non-small-cell lung cancer. Clin Transl Oncol.

[31] Therasse P, Arbuck SG, Eisenhauer EA, Wanders J, Kaplan RS, Rubinstein L, Verweij J, Van Glabbeke M, van Oosterom AT, Christian MC, Gwyther SG (2000). New guidelines to evaluate the response to treatment in solid tumors. European Organization for Research and Treatment of Cancer, National Cancer Institute of the United States, National Cancer Institute of Canada. J Natl Cancer Inst.

[32] Trotti A, Colevas AD, Setser A, Rusch V, Jaques D, Budach V, Langer C, Murphy B, Cumberlin R, Coleman CN, Rubin P (2003). CTCAE v3.0: development of a comprehensive grading system for the adverse effects of cancer treatment. Semin Radiat Oncol.

